# Examining the feasibility of assisted index case testing for HIV case-finding: a qualitative analysis of barriers and facilitators to implementation in Malawi

**DOI:** 10.1186/s12913-024-10988-z

**Published:** 2024-05-09

**Authors:** Caroline J. Meek, Tiwonge E. Mbeya Munkhondya, Mtisunge Mphande, Tapiwa A. Tembo, Mike Chitani, Milenka Jean-Baptiste, Dhrutika Vansia, Caroline Kumbuyo, Jiayu Wang, Katherine R. Simon, Sarah E. Rutstein, Clare Barrington, Maria H. Kim, Vivian F. Go, Nora E. Rosenberg

**Affiliations:** 1 RTI International, Research Triangle Park, NC USA; 2 Gillings School of Global Public Health, University of North Carolina at Chapel Hill, Chapel Hill, NC USA; 3 Kamuzu University of Health Sciences, Blantyre, Malawi; 4 Baylor College of Medicine Children’s Foundation, Lilongwe, Malawi; 5 Department of Medicine, Division of Infectious Diseases, University of North Carolina at Chapel Hill, Chapel Hill, NC USA

**Keywords:** HIV testing and counseling, Index case testing, Assisted partner notification services, Malawi, Implementation science, Health care workers

## Abstract

**Background:**

Assisted index case testing (ICT), in which health care workers take an active role in referring at-risk contacts of people living with HIV for HIV testing services, has been widely recognized as an evidence-based intervention with high potential to increase status awareness in people living with HIV. While the available evidence from eastern and southern Africa suggests that assisted ICT can be an effective, efficient, cost-effective, acceptable, and low-risk strategy to implement in the region, it reveals that feasibility barriers to implementation exist. This study aims to inform the design of implementation strategies to mitigate these feasibility barriers by examining “assisting” health care workers’ experiences of how barriers manifest throughout the assisted ICT process, as well as their perceptions of potential opportunities to facilitate feasibility.

**Methods:**

In-depth interviews were conducted with 26 lay health care workers delivering assisted ICT in Malawian health facilities. Interviews explored health care workers’ experiences counseling index clients and tracing these clients’ contacts, aiming to inform development of a blended learning implementation package. Transcripts were inductively analyzed using Dedoose coding software to identify and describe key factors influencing feasibility of assisted ICT. Analysis included multiple rounds of coding and iteration with the data collection team.

**Results:**

Participants reported a variety of barriers to feasibility of assisted index case testing implementation, including sensitivities around discussing ICT with clients, privacy concerns, limited time for assisted index case testing amid high workloads, poor quality contact information, and logistical obstacles to tracing. Participants also reported several health care worker characteristics that facilitate feasibility (knowledge, interpersonal skills, non-stigmatizing attitudes and behaviors, and a sense of purpose), as well as identified process improvements with the potential to mitigate barriers.

**Conclusions:**

Maximizing assisted ICT’s potential to increase status awareness in people living with HIV requires equipping health care workers with effective training and support to address and overcome the many feasibility barriers that they face in implementation. Findings demonstrate the need for, as well as inform the development of, implementation strategies to mitigate barriers and promote facilitators to feasibility of assisted ICT.

**Trial registration:**

NCT05343390. Date of registration: April 25, 2022.

**Supplementary Information:**

The online version contains supplementary material available at 10.1186/s12913-024-10988-z.

## Introduction

To streamline progress towards its goal of ending AIDS as a public health threat by 2030, the Joint United Nations Programme on HIV/AIDS (UNAIDS) launched a set of HIV testing and treatment targets [1]. Adopted by United Nations member states in June 2021, the targets call for 95% of all people living with HIV (PLHIV) to know their HIV status, 95% of all PLHIV to be accessing sustained antiretroviral therapy (ART), and 95% of all people receiving ART to achieve viral suppression by 2025 [2]. Eastern and southern Africa has seen promising regional progress towards these targets in recent years, and the region is approaching the first target related to status awareness in PLHIV- in 2022, 92% of PLHIV in the region were aware of their status [3]. However, several countries in the region lag behind [4], and as 2025 approaches, it is critical to scale up adoption of evidence-based interventions to sustain and accelerate progress.

Index case testing (ICT), which targets provision of HIV testing services (HTS) for sexual partners, biological children, and other contacts of known PLHIV (“index clients”), is a widely recognized evidence-based intervention used to identify PLHIV by streamlining testing efforts to populations most at risk [5–7]. Traditional approaches to ICT rely on passive referral, in which index clients invite their contacts for testing [5]. However, the World Health Organization (WHO) and the President’s Emergency Plan for HIV/AIDS Relief (PEPFAR) have both recommended assisted approaches to ICT [6, 8–10], in which health care workers (HCWs) take an active role in referral of at-risk contacts for testing, due to evidence of improved effectiveness in identifying PLHIV compared to passive approaches [10–14]. As a result, there have been several efforts to scale assisted ICT throughout eastern and southern Africa in recent years [15–20]. In addition to evidence indicating that assisted ICT can be effective in increasing HIV testing and case-finding [16, 17, 21–24], implementation evidence [25] from the region suggests that assisted ICT can be an efficient [14], acceptable [5, 13, 15, 18, 20, 21, 26], cost-effective [27], and low-risk [21, 22, 24, 28, 29] strategy to promote PLHIV status awareness. However, the few studies that focus on feasibility, or the extent to which HCWs can successfully carry out assisted ICT [25], suggest that barriers exist to feasibility of effective implementation [18–20, 30–32]. Developing informed implementation strategies to mitigate these barriers requires more detailed examination of how these barriers manifest throughout the assisted ICT process, as well as of potential opportunities to facilitate feasibility, from the perspective of the HCWs who are doing the “assisting”.

This qualitative analysis addresses this need for further detail by exploring “assisting” HCWs’ perspectives of factors that influence the feasibility of assisted ICT, with a unique focus on informing development of effective implementation strategies to best support assisted ICT delivery in the context of an implementation science trial in Malawi.

## Methods

### Setting

This study was conducted in the Machinga and Balaka districts of Malawi. Malawi is a country in southeastern Africa in which 7.1% of the population lives with HIV and 94% of PLHIV know their status [4]. Machinga and Balaka are two relatively densely populated districts in the southern region of Malawi [33] with HIV prevalence rates similar to the national average [34]. We selected Machinga and Balaka because they are prototypical of districts in Malawi implementing Ministry of Health programs with support from an implementing partner.

Malawi has a long-established passive ICT program, and in 2019 the country also adopted an assisted component, known as voluntary assisted partner notification, as part of its national HIV testing policy [32]. In Malawi, ICT is conducted through the following four methods, voluntarily selected by the index client: 1) passive referral, in which HCWs encourage the index client to refer partners for voluntary HTS, 2) contract referral, in which HCWs establish an informal ‘contract’ with index clients that agrees upon a date that the HCW can contact the contact clients if they have not yet presented for HTS; 3) provider referral, in which HCWs contact and offer voluntary HTS to contact clients; and 3) dual referral, in which HCWs accompany and provide support to index clients in disclosing their status and offering HTS to their partners [8]. 

While Malawi has one of the lowest rates of qualified clinical HCWs globally (< 5 clinicians per 100,000 people) [35], the country has a strong track record of shifting HTS tasks to lay HCWs, who have been informally trained to perform certain health care delivery functions but do not have a formal professional/para-professional certification or tertiary education degree, in order to mitigate this limited medical workforce capacity [32, 36]. In Malawi, lay HCW roles include HIV Diagnostic Assistants (who are primarily responsible for HIV testing and counseling, including index case counseling) and community health workers (who are responsible for a wider variety of tasks, including index case counseling and contact tracing) [32]. Non-governmental organization implementing partners, such as the Tingathe Program, play a critical role in harnessing Malawian lay HCW capacity to rapidly and efficiently scale up HTS, including assisted ICT [32, 37–39].

### Study design

Data for this analysis were collected as part of formative research for a two-arm cluster randomized control trial examining a blended learning implementation package as a strategy for building HCW capacity in assisted ICT [40]. Earlier work [32] established the theoretical basis for testing the blended learning implementation package, which combines individual asynchronous modules with synchronous small-group interactive sessions to enhance training and foster continuous quality improvement. The formative research presented in this paper aimed to further explore factors influencing feasibility of the assisted ICT from the perspective of HCWs in order to inform development of the blended learning implementation package.

Prior to the start of the trial (October-December 2021), the research team conducted 26 in-depth interviews (IDIs) with lay HCWs at 14 of the 34 facilities included in the parent trial. We purposively selected different types of facilities (hospitals, health centers, and dispensaries) in both districts and from both randomization arms, as this served as a qualitative baseline for a randomized trial. Within these facilities, we worked with facility supervisors to purposively select HCWs who were actively engaged in Malawi’s ICT program from the larger sample of HCWs eligible for the parent trial (had to be at least 18 years old, employed full-time at one of the health facilities included in the parent trial, and involved in counseling index clients and/or tracing their contacts). The parent trial enrolled 306 HCWs, who were primarily staff hired by Tingathe Program to support facilities implementing Malawi’s national HIV program.

### Data collection

IDIs were conducted by three trained Malawian interviewers in a private setting using a semi-structured guide. IDIs were conducted over the phone when possible (*n* = 18) or in-person at sites with limited phone service (*n* = 8). The semi-structured guide was developed for this study through a series of rigorous, iterative discussions among the research team (Additional file 1). The questions used for this analysis were a subset of a larger interview. The interview guide questions for this analysis explored HCWs’ experiences with assisted ICT, including barriers and facilitators to implementation. Probing separately about the processes of counseling index clients and tracing their contacts, interviewers asked questions such as “What is the first thing that comes to mind when you think of counseling index clients/tracing contacts?”, “What aspects do you [like/not like] about…?” and “What do your colleagues say about…?”. When appropriate, interviewers probed further about how specific factors mentioned by the participant facilitate or impede the ICT implementation experience.

The IDIs lasted from 60–90 min and were conducted in Chichewa, a local language in Malawi. Eleven audio recordings were transcribed verbatim in Chichewa before being translated into English and 15 recordings were directly translated and transcribed into English. Interviewers summarized each IDI after it was completed, and these summaries were discussed with the research team routinely.

### Data analysis

The research team first reviewed all of the interview summaries individually and then met multiple times to discuss initial observations, refining the research question and scope of analysis. A US-based analyst (CJM) with training in qualitative analysis used an inductive approach to develop a codebook, deriving broad codes from the implementation factors mentioned by participants throughout their interviews. Along with focused examination of the transcripts, she consulted team members who had conducted the IDIs with questions or clarifications. CJM regularly met with Malawian team members (TEMM, MM, TAT) who possess the contextual expertise necessary to verify and enhance meaning. She used the Dedoose (2019) web application to engage in multiple rounds of coding, starting with codes representing broad implementation factors and then further refining the codebook as needed to capture the nuanced manifestations of these barriers and facilitators. Throughout codebook development and refinement, the analyst engaged in memoing to track first impressions, thought processes, and coding decisions. The analyst presented the codebook and multiple rounds of draft results to the research team. All transcripts and applied codes were also reviewed in detail by additional team members (MJB, DV). Additional refinements to the codebook and results interpretations were iteratively made based on team feedback.

### Ethical clearance

Ethical clearance was provided by UNC’s IRB, Malawi’s National Health Sciences Research Committee, and the Baylor College of Medicine IRB. Written informed consent was obtained from all participants in the main study and interviewers confirmed verbal consent before starting the IDIs.

## Results

Participant characteristics are described in Table 1 below.

**Table 1:** Characteristics of HCWs participating in in-depth interviews^a^

Characteristic	Frequency (%) or Median (IQR)
Age^b^	33 (28, 37)
Sex^b^	
Female	8 (32%)
Male	17 (68%)
HCW cadre^b^	
Community health worker	8 (32%)
HIV diagnostic assistant	17 (68%)
Years of Job Experience^b^	4 (2, 5)
Facility Location	
Rural	18 (69%)
Semi-urban/ Peri-urban/Urban	8 (31%)
Facility type	
Dispensary	4 (15%)
Health Center	16 (62%)
Hospital	6 (23%)

^a^A total of 26 HCWs were sampled from 14 facilities in the study

^b^Missing characteristics data for one HCW

### Factors influencing feasibility of assisted ICT: barriers and facilitators

**Fig. 1:**
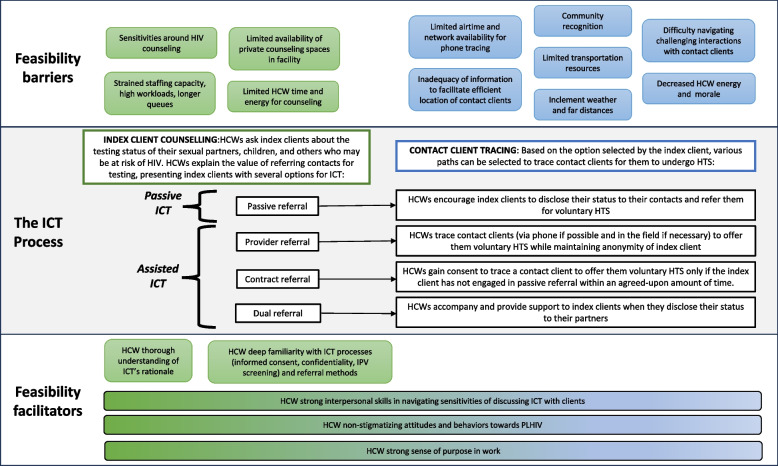
Conceptual diagram mapping feasibility barriers and facilitators to the ICT process

Participants described a variety of barriers and facilitators to feasibility of assisted ICT, manifesting across the index client counseling and contact client tracing phases of the implementation process. Identified barriers included sensitivities around discussing ICT with clients, privacy concerns, limited time for ICT amid high workloads, poor quality contact information, and logistical obstacles to tracing. In addition to these barriers, participants also described several HCW characteristics that facilitated feasibility: ICT knowledge, interpersonal skills, positive attitudes towards clients, and sense of purpose. Barriers and facilitators are mapped to the ICT process in Fig. 1 and described in greater detail in further sections.

### Feasibility barriers

#### Sensitivities around discussing ICT with clients

Participants described ICT as a highly sensitive topic to approach with clients. Many expressed a feeling of uncertainty around how open index clients will be to sharing information about their contacts, as well as how contacts will react when approached for HTS. When asked about difficult aspects of counseling index clients, many HCWs mentioned clients’ hesitance or declination to participate in assisted ICT and share their contacts. Further, several HCWs mentioned that some index clients would provide false contact information. These index client behaviors were often attributed to confidentiality concerns, fear of unwanted status disclosure, and fear of the resulting implications of status disclosure: *“They behave that way because they think you will be telling other people about their status…they also think that since you know it means their life is done, you will be looking at them differently*.” Populations commonly identified as particularly likely to hesitate, refuse, or provide false information included youth (described as “*shy*” “*thinking they know a lot*” and “*difficult to reveal their contacts*”) and newly diagnosed clients *(“it may be hard for them to accept [their HIV diagnosis]”*). One participant suggested that efforts to pair index clients with same-sex HCWs could make them more comfortable to discuss their contacts.

When asked about the first things that come to mind when starting to trace contacts, many participants discussed wondering how they will be received by the contact and preparing themselves to approach the contact. When conducting provider or contract referral, HCWs described a variety of challenging reactions that can occur when they approach a contact for HTS- including delay or refusal of testing, excessive questioning about the identity of the index client who referred them for testing, and even anger or aggression. Particularly mentioned in the context of male clients, these kinds of reactions can lead to stress and uncertain next steps for HCWs: *“I was very tensed up. I was wondering to myself what was going to happen…he was talking with anger.”*

Participants also noted the unique sensitivities inherent in conducting dual referral and interacting with sexual partners of index clients, explaining that HIV disclosure can create acute conflict in couples due to perceived blame and assumptions of infidelity. They recounted these scenarios as particularly difficult to navigate, with high stakes that require high-quality counseling skills: *“sometimes if you do not have good counseling the marriage happens to get to an end.”*. Some participants discussed concern about index client risk of intimate partner violence (IPV) upon partner disclosure: *“they think that if they go home and [disclose their HIV status], the marriage will end right there, or for some getting to a point of [being] beaten.”*

#### Privacy concerns

Participants also reported that clients highly value privacy, which can be difficult to secure throughout the ICT process. In the facility, while participants largely indicated that counseling index clients was much more successful when conducted in a private area, many reported limited availability of private counseling space. One participant described this challenge: “*if I’m counseling an index client and people keep coming into the room…this compromises the whole thing because the client becomes uncomfortable in the end.”* Some HCWs mentioned working around this issue through use of screens, “do-not-disturb” signs, outdoor spots, and tents.

Participants also noted maintaining privacy as a challenge when tracing contact clients in the field, as they sometimes find clients in a situation that is not conducive to private conversations. One participant described: “*we get to the house and find that there are 4, 5 people with our [contact client]…it doesn’t go well…That is a mission gone wrong.*” Participants also noted that HCWs are also often easily recognizable in the community due to their bikes and cars, which exacerbates the risk of compromising privacy. To address privacy challenges in the community, participants reported strategies to increase discretion, including dressing to blend in with the community, preparing an alternate reason to be looking for the client, and offering HTS to multiple people or households to avoid singling out one person.

#### Limited time for ICT amid high workloads

Some participants indicated that strained staffing capacity leads HCWs to have to perform multiple roles, expressing challenges in balancing their ICT work with their other tasks. As one participant described, *“Sometimes it is found that you are assigned a task here at the hospital to screen anyone who comes for blood testing, but you are also supposed to follow up [with] the contacts the same day- so it becomes a problem…you fail to follow up [with] the contacts.”* Some also described being the only, or one of few staff responsible for ICT: *“You’re doing this work alone, so you can see that it is a big task to do it single-handedly.”* The need to counsel each index client individually, as a result of confidentiality concerns, further increases workload for the limited staff assigned to this work. Further, HCWs often described contact tracing in the field as time-consuming and physically taxing, which leaves them less time and energy for counseling. Many HCWs noted the need to hire more staff dedicated to ICT work.

High workloads also resulted in shorter appointments and less time to counsel index clients, which participants reported limits the opportunity for rapport that facilitates openness or probes for detailed information about sexual partners. Participants emphasized the importance of having enough time to meaningfully engage with index clients: *“For counseling you cannot have a limit to say, ‘I will talk to him for 5 min only.’ …That is not counseling then. You are supposed to stay up until…you feel that this [person] is fulfilled.”*. In addition, high workload can reduce the capacity of HCWs to deliver quality counseling: *“So you find that as you go along with the counseling, you can do better with the first three clients but the rest, you are tired and you do short cuts.”*

High workloads also lead to longer queues, which may deter clients from coming into the clinic or cause them to leave before receiving services: *“Sometimes because of shortage of staff, it happens that you have been assigned a certain task that you were supposed to do but at the same time there are clients who were supposed to be counseled. As a result, because you spent more time on the other task as a result you lose out some of the clients because you find that they have gone.”* In response to long queues, several participants described ‘fast-tracking’ contact clients who come in for HTS in effort to maximize case-finding by prioritizing those who have been identified as at risk of HIV.

#### Poor quality contact information

Participants repeatedly discussed the importance of eliciting accurate information about a person’s sexual partners, including where, when, and how to best contact them. As one participant said, “*Once the index has given us the wrong information then everything cannot work, it becomes wrong…if he gives us full information [with] the right details then everything becomes successful and happens without a problem.*” Adequate information is a critical component of the ICT process, and incorrect or incomplete information delays or prevents communication with contact clients.

Inadequate information, which can include incorrect or incomplete names, phone numbers, physical addresses, and contextual details, can arise from a variety of scenarios. Most participants mentioned index clients providing incorrect information as a concern. This occurred either intentionally to avoid disclosure or unintentionally if information was not known. Poor quality contact information also results from insufficient probing and poor documentation, which is often exacerbated by aforementioned HCW time and energy constraints. In one participant’s words, *“The person who has enlisted the contact…is the key person who can make sure that our tracing is made easy.”* Participants noted the pivotal role of the original HCW who first interacts with the index client in not only eliciting correct locator information but also eliciting detailed contextual information. For example, details about a contact client’s profession are helpful to trace the client at a time when they will likely be at home. Other helpful information included nicknames, HIV testing history, and notes about confidentiality concerns.

#### Logistical obstacles to tracing

Some contact clients are reached by phone whereas others must be physically traced in the community. Some participants reported difficulty with tracing via phone, frequently citing network problems and lack of sufficient airtime allocated by the facility. Participants also reported that some clients were unreachable by phone, necessitating physical tracing. Physically tracing a contact client requires a larger investment of resources than phone tracing, especially when the client lives at a far distance from the clinic. Participants frequently discussed having to travel far distances to reach contact clients, an issue some saw as exacerbated by people who travel to clinics at far distances due to privacy concerns.

While most participants reported walking or biking to reach contact clients in the community, some mentioned using a motorcycle or Tingathe vehicle. However, access to vehicles is often limited and these transportation methods require additional expenses for fuel. Walking or biking was also reported to expose HCWs to inclement weather, including hot or rainy seasons, and potential safety risks such as violence.

Participants reported that traveling far distances can be physically taxing and time-consuming, sometimes rendering them too tired or busy to attend to other tasks. Frequent travel influenced HCW morale, particularly when a tracing effort did not result in successfully recruiting a contact client. Participants frequently described this perception of wasted time and energy as “*painful*”, with the level of distress often portrayed as increasing with the distance travelled. As one HCW said, *“You [can] find out that he gave a false address. That is painful because it means you have done nothing for the person, you travelled for nothing.”*

HCWs described multiple approaches used to strategically allocate limited resources for long distances. These approaches included waiting to physically trace until there are multiple clients in a particular area, reserving vehicle use for longer trips, and coordinating across HCWs to map out contact client locations. HCWs also mentioned provision of rain gear and sun protection to mitigate uncomfortable travel. Another approach involved allocating contact tracing to HCWs based in the same communities as the contact clients.

### Feasibility facilitators

#### HCW knowledge about ICT

Participants reported that HCWs with a thorough understanding of ICT’s rationale and purpose can facilitate client openness. Clients were more likely to engage with HCWs about assisted ICT if they understood the benefits to themselves and their loved ones. One HCW stated, *“If the person understands why we need the information, they will give us accurate information.”*

Participants also discussed the value of deep HCW familiarity with ICT procedures and processes, particularly regarding screening clients for IPV and choosing referral method. One participant described the importance of clearly explaining various referral methods to clients: *“So…people come and choose the method they like…when you explain things clearly it is like the index client is free to choose a method which the contact can use for testing”.* Thorough knowledge of available referral methods allows HCWs to actively engage with index clients to discuss strategies to refer contacts in a way that fits their unique confidentiality needs, which was framed as particularly important when IPV is identified as a concern. Multiple participants suggested the use of flipcharts or videos, saying these would save limited HCW time and energy, fill information gaps, and provide clients with a visual aid to supplement the counseling. Others suggested recurring opportunities for training, to continuously “refresh” their ICT knowledge in order to facilitate implementation.

#### HCW interpersonal skills

In addition, HCWs’ ability to navigate sensitive conversations about HIV was noted as a key facilitator of successful implementation. Interpersonal skills were mentioned as mitigating the role’s day-to-day uncertainty by preparing HCWs to engage with clients, especially newly diagnosed clients: “*I need to counsel them skillfully so that they understand what I mean regardless that they have just tested positive for HIV.”*

When discussing strategies to build HCW skills in counseling index clients and tracing contact clients, participants suggested establishing regular opportunities to discuss challenges and share approaches to address these challenges: “*I think that there should be much effort on the [HCWs] doing [ICT]. For example, what do I mean, they should be having a meeting with the facility people to ask what challenges are you facing and how can we end them?”.* Another participant further elaborated, saying *“We should be able to share experiences with our [colleagues] so that we can all learn from one another. And also, there are other people who are really brilliant at their job. Those people ought to come visit us and see how we are doing. That is very motivating.”*

#### HCW non-stigmatizing attitudes and behaviors

Participants also highlighted the role of empathy and non-judgement in building trust with clients: “*Put yourself in that other person’s shoes. In so doing, the counseling session goes well. Understanding that person, that what is happening to them can also happen to you.*”. Participants viewed trust-building as critical to facilitating client comfort and openness: *“if they trust you enough, they will give you the right information.”* Further, participants associated HCW assurance of confidentiality with promoting trust and greater information sharing: “*Also assuring them on the issue of confidentiality because confidentiality is a paramount. If there will not be confidentiality then the clients will not reveal.”*

#### HCW sense of purpose

Lastly, several participants reported that a sense of purpose and desire to help people motivated them to overcome the challenges of delivering assisted ICT. One participant said, “*Some of these jobs are a ministry. Counseling is not easy. You just need to tell yourself that you are there to help that person.*” Many seemed to take comfort in the knowledge that their labors, however taxing, would ultimately allow people to know their status, take control of their health, and prevent the spread of HIV. Participants framed the sense of fulfillment from successful ICT implementation as a mitigating factor amidst challenges: “*If [the contact client] has accepted it then I feel that mostly I have achieved the aim of being in the health field…that is why it is appealing to me*”.

## Discussion

Participants described a variety of barriers to assisted ICT implementation, including sensitivities around discussing ICT with clients, privacy concerns, limited time for ICT amid high workloads, poor quality contact information, and logistical obstacles to tracing. These barriers manifested across each step of the process of counseling index clients and tracing contacts. However, participants also identified HCW characteristics and process improvements that can mitigate these barriers.

Further, participants’ descriptions of the assisted ICT process revealed the intimately interconnected nature of factors that influence feasibility of assisted ICT. Sensitivities around HIV, privacy limitations, time constraints, and HCW characteristics all contribute to the extent to which counseling index clients elicits adequate information to facilitate contact tracing. Information quality has implications for HCW capacity, as inadequate information can lead to wasted resources, including HCW time and energy, on contact tracing. The opportunity cost of wasted efforts, which increases as the distance from which the contact client lives from the clinic increases, depletes HCW morale. The resulting acceleration of burnout, which is already fueled by busy workloads and the inherent uncertainty of day-to-day ICT work, further impairs HCW capacity to effectively engage in quality counseling that elicits adequate information from index clients. This interconnectedness suggests that efforts to mitigate barriers at any step of the assisted ICT process may have the potential to ripple across the whole process.

Participants’ descriptions of client confidentiality and privacy concerns, as well as fear of consequences of disclosure, align with previous studies that emphasize stigma as a key barrier to assisted ICT [15, 18–20, 30, 31] and the overall HIV testing and treatment cascade [41]. Our findings suggest that anticipated stigma, or the fear of discrimination upon disclosure [42], drives several key barriers to feasibility of assisted ICT implementation. Previous studies also highlight the key role of HCWs in mitigating barriers related to anticipated stigma; noting the key role of HCW ICT knowledge, interpersonal skills, and non-stigmatizing attitudes/behaviors in securing informed consent from clients for ICT, tailoring the referral strategy to minimize risk to client confidentiality and safety, building trust and rapport with the client, and eliciting accurate contact information from index clients to facilitate contact tracing [18–20, 30].

Our findings also reflect previous evidence of logistical challenges related to limited time, space, and resources that can present barriers to feasibility for HCWs [18–20, 30, 31]. Participants in the current study described these logistical challenges as perpetuating HCW burnout, making it harder for them to engage in effective counseling. Cumulative evidence of barriers across different settings (further validated by this study) suggests that assisted ICT implementation may pose greater burden on HCWs than previously thought [7]. However, our findings also suggest that strategic investment in targeted implementation strategies has the potential to help overcome these feasibility barriers.

In our own work, these findings affirmed the rationale for and informed the development of the blended learning implementation package tested in our trial [40, 43]. Findings indicated the need for evidence-based training and support to promote HCW capacity to foster facilitating characteristics. Participants discussed the value of "refresher" opportunities in building knowledge, as well as the value of learning from other’s experiences. The blended learning implementation package balances both needs by providing time for HCWs to master ICT knowledge and skills with a combination of asynchronous, digitally delivered content (which allows for continuous review as a "refresher") and in-person sessions (which allow for sharing, practicing, and feedback). Our findings also highlight the value of flexible referral methods that align with the client’s needs, so our training content includes a detailed description of each referral method process. Further, our training content emphasizes client-centered, non-judgmental counseling as our findings add to cumulative evidence of stigma as a key barrier to assisted ICT implementation [41].

In addition, participants frequently mentioned informal workarounds currently in use to mitigate barriers or offered up ideas for potential solutions to try. Our blended learning implementation package streamlines these problem-solving processes by offering monthly continuous quality improvement sessions at each facility in our enhanced arm. These sessions allow for structured time to discuss identified barriers, share ideas to mitigate barriers, and develop solutions for sustained process improvement tailored to their specific setting. Initial focus areas for continuous quality improvement discussions include use of space, staffing, allocation of airtime and vehicles, and documentation, which were identified as barriers to feasibility in the current study.

Our study provides a uniquely in-depth examination of HCWs’ experiences implementing assisted ICT, exploring how barriers can manifest and interact with each other at each step of the process to hinder successful implementation. Further, our study has a highly actionable focus on informing development of implementation strategies to support HCWs implementing assisted ICT. Our study also has limitations. Firstly, while our sole focus on HCWs allowed for deeper exploration of assisted ICT from the perspective of those actually implementing it on the ground, this meant that our analysis did not include perspectives of index or contact clients. In addition, we did not conduct sub-group analyses as interpretation of results would be limited by our small sample size.

## Conclusions

Assisted ICT has been widely recognized as an evidence-based intervention with high promise to increase PLHIV status awareness [5–7, 10, 12–21, 23, 24, 26–29], which is important as countries in eastern and southern Africa strive to reach global UNAIDS targets. Study findings support cumulative evidence that HCWs face a variety of feasibility barriers to assisted ICT implementation in the region; further, the study’s uniquely in-depth focus on the experiences of those doing the “assisting” enhances understanding of how these barriers manifest and informs the development of implementation strategies to mitigate these barriers. Maximizing assisted ICT’s potential to increase HIV testing requires equipping HCWs with effective training and support to address and overcome the many feasibility barriers they face in implementation. Findings demonstrate the need for, as well as inform the development of, implementation strategies to mitigate barriers and promote facilitators to feasibility of assisted ICT.

### Supplementary Information

**Supplementary Material 1. **

## Data Availability

Qualitative data on which this analysis is based, as well as data collection materials and codebooks, are available from the last author upon reasonable request. The interview guide is included as an additional file.

## Abbreviations

AIDS: Acquired Immunodeficiency Syndrome
ART: Antiretroviral Therapy
HCW: Health Care Worker
HIV: Human Immunodeficiency Virus
HTS: HIV Testing Services
ICT: Index Case Testing
IDI: In-Depth Interview
IPV: Intimate Partner Violence
IRB: Institutional Review Board
PEPFAR: President’s Emergency Plan for HIV/AIDS Relief
PLHIV: People Living With HIV
UNAIDS: Joint United Nations Programme on HIV/AIDS
WHO: World Health Organization
