# Supplementary material for: Examining the feasibility of assisted index case testing for HIV case-finding: a qualitative analysis of barriers and facilitators to implementation in Malawi
Source: BMC Health Serv Res. 2024 May 9;24:606. doi: 10.1186/s12913-024-10988-z (PMC11080127; doi:10.1186/s12913-024-10988-z)
Supplement: Supplementary file 1 — Supplementary Material 1. [file 12913_2024_10988_MOESM1_ESM.docx]

# **PRACTICE Study: In-Depth Interview (IDI) Guide (English)**

**Date |___|___| / |___|___|___|/ |___|___| _(DD/MMM/YY)_**

**Start time |___|___|:|___|___| End time |___|___|:|___|___|**

**Facilitator ID |___|___|___|___|___|**

**Facility ID |___|___| Health worker ID |___|___|___|___|___|**

**Wave** _(1)_ Pre ****_(2)_ Post 1 ****_(3)_ Post 2

**Study arm** _(0)_ Standard of care ****_(1)_ Enhanced

# **Introduction**

Thank you all for meeting with me today. We appreciate your time. We have invited you to participate in this discussion to help us learn about your experiences with Malawi’s index case testing (ICT) and voluntary assisted partner notification (VAPN) program. We expect this conversation will take approximately one hour. Before we get started, we wanted to set some ground rules for this discussion:

- I hope you will feel open and share your thoughts. There are no right or wrong answers. We want to hear about your honest opinions, even if they are not positive.
- We will be audio-recording this information. Although we are recording, your information will be de-identified and will be kept confidential. We will only share what health workers said overall, but not what any individual person said. We will also not share what you say with your supervisors. It is ok to be critical even of them.
- We also want to protect other peoples’ confidentiality. Please do not share the names of any patients, health workers, or health facilities. You can refer to “a colleague” or “this facility” but please do not use names.

What questions or concerns do you have for me before we begin?

I am going to turn on the audio recorder now. Is this ok? ***[Turn audio recording on]***

Today is date. This is time. This interview is being conducted by facilitator ID XXXXX. This is an interview with ID XXXXX conducted at facility ID XXX in the XX arm. Before we begin, I would like to confirm that you have been consented and agree to participate in this study. Is it ok if we begin now?

## **I. Experiences with standard of care training *(pre only)***

1. I would like to start by asking you about any trainings you have attended on index case testing (ICT) and voluntary assisted partner notification (VAPN). I would like to start by learning about any in-person training you received on index testing and contact tracing.

2) In your own words, how can you describe your experience with your most recent training on ICT/VAPN?

*Probe on delivery modality, facilitation skills, and content.*

3) What were the things about the training that you liked most?

*Probe on the reasons why they liked these aspects.*

4) What are the things you liked least or found the most challenging?

*Probe on the reasons why they found these things challenging.*

5) What were the main things that you learned?

*Probe for additional areas and/or additional details.*

6) Will you describe the things from the training that you apply in your work?

*Probe for additional areas and/or additional details.*

7) What things during the training might have helped you learn the material better?

*Probe on role modeling, practicing, and receiving feedback.*

8) Part of training is learning new material, but many health workers enjoy other benefits to training. Tell me about any other aspects of training that are appealing to you and why?

*Probe on seeing colleagues from other facilities, traveling away from home, allowances, different food, and having time away from work.*

9) Other than the new material, there may be aspects of training that are unappealing. Tell me about the aspects of training that are unappealing to you and why?

*Probe on seeing colleagues from other facilities, traveling away from home, allowances, different food, and having time away from work.*

**II. Anticipating acceptability of the blended learning *(pre)***

In this study, some health care workers will receive additional ongoing support through a “blended learning model.” The blended learning approach refers to the use of both digital and face to face training sessions. Mostly you would do the *digital* training on your own on a tablet. You would have the opportunity to be refreshed on the main concepts covered in the original ICT/VAPN training. You would also watch other health care workers deliver index testing and contact tracing to clients and learn what they did well and what they could have done better. In the *face-to-face* portion, you would have the opportunity to practice with your colleagues and receive feedback from a facilitator. We will now discuss your views regarding the use of the blended learning approach to enhance the training for the ACT programme.

1) What is your understanding regarding how the blended learning package is? What questions do you have?

2) Now would you please share with me your feelings about the use of the digital portion.

- 1. What do you think might be good about this part of the approach? *(Probe more why they feel the digital learning is good, including its usefulness and their confidence)*
  2. What are some of the challenges or burdens you are anticipating facing with the use of digital learning?

*Probes on why this is so.*

3) Now would you share with me your feelings about the face-to-face portion?

a. What do you think is potentially good about this part of the approach?

*Probe more on why they feel the face-to-face portion is good.*

b. What potential challenges would you anticipate with the face-to-face portion?

*Probe more why.*

4) Now I want to ask you about how and where the blended learning might be delivered.

1. What do you perceive as the benefits or profits of participating in this blended learning package if this were delivered near your facility with only colleagues from your facility?

*Probe each item including allowances, time away from work, travel, seeing colleagues from other places? What else? what do others think?)*

1. How do you think your colleagues might react if you were released from one of your work shifts and given time to work on the digital learning at your facility?

*Probes: why would they react that way?*

1. How might they react if a group of you completed the digital learning all together at your facility over the weekend?

*Probe on the potential benefits and downsides.*

1. Given that you have ever received an allowance for Tingathe training, how important is it to receive an allowance for ongoing training?

*Probe on the potential benefits and downsides.*

**III. Experiences with enhanced training *(post 1, enhanced arm only)***

I would like to start by asking you about the blended learning training you received around voluntary assisted partner notification (VAPN) in the last few weeks. Some of this training was self-guided and, on a tablet, and some of this was conducted in small groups.

1) In your own words, can you describe your experience with the self-guided portions of the training?

*Probe on delivery modality, role-model videos, and content.*

2) In your own words, can you describe your experience with the small group portion of the training?

*Probe on delivery modality, facilitation skills, and content.*

3) Overall, tell me about the parts of the training that you liked most.

*Probe on the reasons why they liked these aspects.*

4) Overall, tell me about the parts of the training that you liked least or found the most challenging?

*Probe on the reasons why they found these things challenging.*

5) What were the main things that you learned (or improved on) in the training?

*Probe for additional areas and/or additional details*

6) Tell me about the things from the training that you apply in your work.

*Probe for additional areas and/or additional details*

7) What do you think might have helped you learn the material better?

*Probe on role modeling, practicing, and receiving feedback.*

**IV. Experiences with enhanced training *(post 2, enhanced arm only)***

I would like to start by asking you about the blended learning training you received around voluntary assisted partner notification (VAPN) almost a year ago. Some of this training was self-guided and on a tablet and some of this was conducted in small groups.

1) Tell me about the things from the training that you still apply in your work.

2) What do you think might have helped you learn the material better?

*Probe on role modeling, practicing, and receiving feedback.*

**V.** **Acceptability and feasibility of counseling index clients**

I want to get a sense of your work counseling index clients. Is this something you do sometimes?

1. Tell me about your work counseling index clients.

*Probe on the frequency, nature, and location/population.*

2. What are the first things that come to mind when thinking about counseling index clients?

*Probe on their responses.*

3. What aspects of counseling index clients do you enjoy most? Tell me more about this.

4. What aspects of counseling index clients do you dislike or find challenging or unappealing?

*Probe on the reasons why they found these things challenging.*

5. What types of index clients are the most difficult to counsel? Tell me about this.

6. Can you tell us about the space you have to conduct index counseling?

*Probe on issues of privacy.*

7. Can you share about the amount of time you have to conduct index counseling?

*Probe on issues of time.*

8. Are there times when you are expected to screen or counsel index clients, but do not? If so, what are some of the reasons you do not do it?

9. What do you think should be done to make index counseling easier or more appealing?

10. What do your coworkers say is challenging about index counseling?

11. *(Post 1 and 2 only)* What activities have there been at your facility to improve index counseling?

*Probe on the frequency and nature of these activities and perceptions around their usefulness.*

12. *(Post 1 and 2 only)* Thinking about the last few weeks (post 1)/year (post 2), what has changed regarding index counselling?

**VI. Acceptability and feasibility of tracing and counseling contacts**

Now I would like you to think about your experience tracing contacts. By this, we mean conducting phone or physical tracing in the community to find the sexual partners, children, household members, or social contacts of index clients.

1. Tell me about your work tracing contacts.

*Probe on the frequency, type, and nature of tracing contacts.*

2. What are the first things that come to mind when thinking about tracing contacts?

*Probe on their responses.*

3. What aspects of tracing contacts do you enjoy? Tell me about this.

4. What aspects of tracing contacts do you dislike or find challenging or unappealing?

5. What contacts are the most difficult to find and/or counsel? Tell me about this.

6. Can you tell us about the resources you have for phone tracing? What challenges do you face in this area?

*Probe on issues of call quality, air time.*

7. Can you talk about the transportation surrounding physical tracing? What challenges do you face in this area?

*Probe on issues of locating contacts.*

8. Are there times when you are expected to conduct contact tracing but do not? If so, what are some of the reasons that stop you from conducting contact tracing?

9. What do you think can be done to make contact tracing easier or more appealing?

10. What do your coworkers say is challenging about tracing contacts?

11. *(Post 1 and 2 only)* What activities have there been at your facility to improve contact tracing?

*Probe on the frequency and nature of these activities and perceptions around their usefulness.*

12. *(Post 1 and 2 only)* Thinking about the last few weeks (post 1)/year (post 2), what has changed regarding contact tracing?

## **VII. Provider attitudes**

## In your job, you and your coworkers work with many different types of clients and patients. I want us to discuss what you might see or observe others saying or doing regarding index clients and contacts, many of whom are people living with HIV.

## 1. How do health workers at your facility look at people living with HIV?

## 2. Generally, how are index clients and their contacts treated by health care workers at your health facility?

## 3. Have you ever observed health care workers treating certain types of index clients or contacts poorly? Can you describe these observations?

## 4. Do you ever observe health care workers treating certain types of index clients or contacts especially well? Can you describe these observations?

**VIII. Closing questions and remarks**

1. What else would you like me to know about counseling index clients or tracing their contacts or about the trainings you have received?

This ends our discussion today. Thank you for your time. We really appreciate the time you took to participate in this discussion today. We will now end the audio recording.

***[Turn audio recording off]***
